# Genome features and antimicrobial resistance of *Trueperella pyogenes* isolated from deceased farmed white-tailed deer (*Odocoileus virginianus*) in Florida

**DOI:** 10.1128/spectrum.00119-26

**Published:** 2026-06-30

**Authors:** An-Chi Cheng, Ting Liu, Austin C. Surphlis, John A. Lednicky, Samantha M. Wisely, Kuttichantran Subramaniam, Kwangcheol C. Jeong, Juan M. Campos Krauer

**Affiliations:** 1 Department of Large Animal Clinical Sciences, College of Veterinary Medicine, University of Florida 70122, Gainesville, Florida, USA; 2 Department of Animal Sciences, College of Agriculture and Life Sciences, University of Florida 539029, Gainesville, Florida, USA; 3 Emerging Pathogens Institute, University of Florida 145775, Gainesville, Florida, USA; 4 Department of Infectious Diseases and Immunology, College of Veterinary Medicine, University of Florida 70122, Gainesville, Florida, USA; 5 Department of Environmental and Global Health, College of Public Health and Health Professions, University of Florida 271714, Gainesville, Florida, USA; 6 Department of Wildlife Ecology and Conservation, Institute of Food and Agricultural Sciences, University of Florida 53701, Gainesville, Florida, USA; Houston Methodist Hospital, Houston, Texas, USA

**Keywords:** *Trueperella pyogenes*, white-tailed deer, deer farming, antimicrobial resistance, virulence factor

## Abstract

*Trueperella pyogenes* is a gram-positive, facultatively anaerobic bacterium known to cause infections in both domestic and wild animals, including white-tailed deer (WTD; *Odocoileus virginianus*). However, limited studies have focused on its impact on farmed WTD. In this study, we aimed to explore the demographic features, antimicrobial resistance (AMR) profiles, and virulence factors of *T. pyogenes* isolated from farmed WTD in Florida. Between 2016 and 2023, we managed 831 deceased farmed WTD cases. Specimens from 715 animals were subjected to bacterial culture. *T. pyogenes* was isolated from 147 (20.6%) animals, ranking as the second most frequently isolated bacterial pathogen. All *T. pyogenes*-infected animals exhibited pneumonia. *T. pyogenes* infection cases were notably more prevalent during summer and fall. Between September 2020 and May 2022, all available *T. pyogenes* isolates (*n* = 37) recovered from various specimen types from 30 diseased deer were included and analyzed using whole-genome sequencing and phylogenetic approaches. The results revealed substantial single-nucleotide polymorphism (SNP) diversity within the core genomes of the *T. pyogenes* strains. Antimicrobial resistance profiling showed that tetracycline resistance genes, particularly *tetW*, predominated, while genes associated with resistance to aminoglycosides, sulfonamides, and other tetracyclines were detected sporadically. Genes for several virulence factors were also identified, including pyolysin (*plo*), collagen-binding protein (*cbpA*), and various adhesion molecules. These findings highlight the importance of disease management strategies, including surveillance and targeted antimicrobial use, to mitigate *T. pyogenes* transmission in farmed WTD. Broader surveillance using non-postmortem clinical specimens and culture-independent approaches is needed to better understand its epidemiology.

**IMPORTANCE:**

*Trueperella pyogenes* is an opportunistic pathogen that has likely been underrecognized in its role in white-tailed deer health. Our demographic and genomic analysis of *T. pyogenes* isolated from deceased farmed white-tailed deer in Florida identified seasonal patterns, frequent association with pneumonia, and substantial genomic diversity among isolates. These findings highlight the need for enhanced surveillance, improved diagnostic approaches, and preventive measures to better understand and manage *T. pyogenes* infections in farmed and free-ranging cervid populations. By characterizing the demographic and genomic features of *T. pyogenes* in this understudied host system, this study provides a foundation for future research on pathogen ecology, disease surveillance, and cervid health management, including monitoring in settings where farmed deer may interact with surrounding wildlife.

## INTRODUCTION

*Trueperella pyogenes* is a gram-positive, facultatively anaerobic bacterium that has a single chromosome with a genome size of approximately 2.3 Mbp (1, 2). It belongs to the family *Actinomycetaceae* (3). This bacterium was previously named *Corynebacterium pyogenes*, *Actinomyces pyogenes* (4, 5), and *Arcanobacterium pyogenes* (6). *Trueperella pyogenes* is an opportunistic pathogen, responsible for various clinical manifestations, such as mastitis, metritis, pneumonia, and suppurative abscesses, affecting multiple organs in animal hosts (Tables 1 and 2).

**TABLE 1:** Overview of the geographic distribution, clinical manifestations, and specimen types for *Trueperella pyogenes* strains obtained from captive cattle globally, based on selected references

Locations	Clinical signs	Specimen types	References
Canada (AB)	Morbid or bovine respiratory disease	Nasal swabs, lungs, laryngeal/tracheal swabs, hearts/pericardium, joint fluid	(7)
USA (NY)	Puerperal metritis or healthy	Uterine secretion	(8)
USA (NY)	Cytologic endometritis, purulent uterine lavage, or purulent vaginal discharge	Uterine lavage specimens	(9)
USA (NY)	Purulent vaginal discharge	Intrauterine specimens with cytobrush	(10)
USA (WA)	Metritis, clinical endometritis, or subclinical endometritis	Uterine secretion with cytobrush	(11)
USA	Liver abscess	Liver abscesses	(12)
USA (FL)	Metritis or healthy	Vaginal mucus	(13)
USA (KS, ND)	N/A	Abscess, fluid, foot, joint, liver, lung, milk, peri rumen, uterus, vaginal swab, wound	(14)
Brazil	Mastitis, abscesses, pneumonia, lymphadenitis, septicemia, or encephalitis	Milk, abscesses, broncho-alveolar lavage fluid, lymph nodes, blood, cerebrospinal fluid, uterus secretion, semen, various tissues	(15)
Denmark	Normal lochia or brown and fetid vaginal discharge	Endometrial tissues	(16)
Denmark	Pyometra	Endometrial tissues	(17)
Netherlands	Udder cleft dermatitis	Lesion tissues, lesion swabs	(18)
Belgium	Abortion or perinatal mortality	Fetal or calf abomasal content, lungs	(19)
Germany	Metritis, endometritis, fertility problems, pneumonia, or septicemia	Milk, uteruses, placentas, cervix or vaginal discharge, lungs, kidneys, urine, feces, abscesses	(20)
Poland, Belarus	Clinical mastitis	Inflamed secretion	(21)
Switzerland	Intramural myocardial abscess	Intramural myocardial abscesses	(22)
Austria	Subclinical endometritis, clinical endometritis, or healthy	Intrauterine specimens with cytobrush	(23)
Spain	Mastitis	Udder lesions, lymph nodes	(24)
Portugal	Normal puerperium or clinical metritis	Uterine swabs	(25)
Turkey	N/A^*a*^	Milk, vaginal fluid, abscesses, pleural fluid, aborted fetuses, synovial fluid	(26)
China	Clinical or subclinical mastitis	Milk	(27)
China	Endometritis	Uterine secretion	(28)
China	Bovine respiratory disease complex	Lungs	(29)

^*a*^ N/A not available.

**TABLE 2:** Geographic locations, clinical signs, captivity status, and specimen types for *Trueperella pyogenes* strains from free-ranging and captive artiodactyl species worldwide, compiled from selected references

Family	Species	Location	Clinical sign	Captivity status	Specimen type	Reference
*Bovidae*						
	Chamois (*Rupicapra pyrenaica*)	Spain	Pyogenic arthritis	Free-ranging	Forelimb	(30)
	Sheep	Spain	Lymphadenitis	Captive	Pus from subcutaneous abscesses	(31)
	Goat	Spain	Lymphadenitis	Captive	Pus from subcutaneous abscesses	(31)
	European bison (*Bison bonasus*)	Poland	Balanoposthitis, abscesses in internal organs	Free-ranging	Tissues with pathological lesions	(32)
	Sannen goat	Iran	Osteomyelitis, pneumonia	Captive	Mandible aspirates	(33)
	Cashmere (Raini) goat	Iran	Bronchopneumonia, hepatitis, myocardial abscesses	Captive	Abscess from lungs, livers, and hearts	(34)
	Dorper sheep	South Africa	Ulcerative balanitis and vulvitis	Captive	Genital swabs	(35)
	Blackbuck (*Antilope cervicapra*)	Australia	Mandibular osteomyelitis, pneumonia	Captive	Mandibular abscesses, pleural effusion, lung parenchyma	(36)
*Camelidae*						
	Camel (*Camelus dromedarius*)	Lybia	Lymphadenitis	Nomadic	Lymph nodes	(37)
	Camel (*Camelus dromedarius*)	Egypt	Pneumonia	Captive	Lungs	(38)
	Camel (*Camelus dromedarius*)	Jordan	Dermatophilosis, lymphadenitis	Captive	Pus, skin scabs	(39)
	Camel (*Camelus dromedarius*)	Saudi Arabia	Repeat breeding syndrome	Captive	Cervical swabs	(40)
*Cervidae*						
	WTD (*Odocoileus virginianus*)	Canada	Necrobacillosis	Captive	Lesion swabs	(41)
	WTD (*Odocoileus virginianus*)	Canada, USA	Intracranial abscesses	Free-ranging	Skulls	(42)
	WTD (*Odocoileus virginianus*)	USA (PA, OH, MO)	Pneumonia	Captive	Lungs	(43)
	WTD (*Odocoileus virginianus*)	USA (MD)	Intracranial abscess	Free-ranging	Antler base and nasopharyngeal mucosa swabs	(44)
	WTD (*Odocoileus virginianus*)	USA (MD)	Intracranial abscessation, suppurative meningitis, or none	Free-ranging	Head and nasal swabs	(45)
	WTD (*Odocoileus virginianus*)	USA (WI)	Pneumonia, septicemia, necrotizing stomatitis, epicarditis/pericarditis, abscess	Captive	Abscesses	(46)
	WTD (*Odocoileus virginianus*)	USA (ND)	Bronchopneumonia	Captive	Lungs	(47)
	WTD (*Odocoileus virginianus*)	USA (GA)	Cranial abscess or grossly healthy	Free-ranging	Forehead, lingual, and nasal swabs	(48)
	Key deer (*Odocoileus virginianus* clavium)	USA (FL)	Chronic purulent infections in cranial and other regions	Free-ranging	Abscess swabs	(49)
*Cervidae*						
	Mule deer (*Odocoileus hemionus*)	USA (WY)	Keratoconjunctivitis	Free-ranging	Lesions	(50)
	Reindeer (*Rangifer tarandus tarandus*)	Norway	Keratoconjunctivitis	Semidomesticated	Conjunctival sac swabs	(51)
	Southern pudu (*Pudu puda*)	UK	Uterine prolapse, septicemia, metritis	Captive	Lungs, livers, uterine exudates	(52)
	Roe deer (*Capreolus capreolus*)	Germany	Brain abscess	N/A	Brain abscesses	(53)
	Fallow deer (*Dama dama*)	Spain	Purulent foot infection	Free-ranging	Coronary band tissues	(54)
	Forest musk deer (*Moschus berezovskii*)	China	Surface or internal abscesses	Captive	Necks, livers, lungs, musk pods, faces	(55)
*Giraffidae*						
	Okapi (*Okapia johnstoni*)	USA (CA)	Fibrinous pleuritis	Captive	Pleural fluid, fibrous adhesions	(56)
	Okapi (*Okapia johnstoni*)	Germany	N/A	Captive	Vaginal discharge	(57)
*Suidae*						
	Pig (*Sus domesticus*)	USA (NC)	Scours, pneumonia	Captive	Gastric mucosa	(58)
	Pig (*Sus domesticus*)	Brazil	Abscess	Captive	Abscesses	(15)
	Pig (*Sus domesticus*)	Brazil	Pneumonia, cervical abscesses	Captive	Lungs, cervical abscesses	(59)
	Pig (*Sus domesticus*)	Spain	Pneumonia, endocarditis, arthritis, lymphadenitis, abscess, pyogranuloma-like lesions	Captive	Lungs, lymph nodes, joints, livers, hearts, spleens, abscesses, brains, kidneys, tonsils	(60)
	Pig (*Sus domesticus*)	Poland	Abscess, pneumonia	Captive	Abscesses	(61)
	Pig (*Sus domesticus*)	Poland	Purulent lesions or abscesses in various tissues, pneumonia	Captive	Abscesses	(62)
	Pig (*Sus domesticus*)	China	Respiratory clinical signs, purulent lesions in lungs	Captive	Lungs	(63)
	Pig (*Sus domesticus*)	China	Pneumonia, severe abdominal effusion	Captive	Lungs	(64)

*Trueperella pyogenes* exhibits a wide host range, affecting a variety of mammals and non-mammals. Cattle are the most extensively studied host, with mastitis, metritis, and abscess formation being the most frequently observed clinical signs (Table 1). The pathogen has also been reported in numerous wild and captive artiodactyl species worldwide (Table 2). It is important to note that *T. pyogenes* infections have been identified in members of the *Cervidae* family, one of the most widely distributed taxa across the United States of America (USA). These infections have been associated with conditions such as abscesses, lumpy jaw, and pneumonia in deer hosts (Table 2). While *T. pyogenes* is relatively uncommon as a clinical pathogen in companion animals, it has been documented in species such as rabbits (*Oryctolagus cuniculus*) (65) and cats (15, 66, 67). This bacterial species has also been identified in birds and reptiles, such as American fantailed pigeons in India with hepatic abscesses (68), a bearded dragon (*Pogona vitticeps*) with septicemia, a gecko with enteritis and septicemia (69), turkeys in the North Central USA exhibiting lameness and osteomyelitis (70), and a zoo-kept royal python (*Python regius*) in Germany (57). In humans, *T. pyogenes* can cause pronounced infections, particularly in individuals with compromised immune systems, leading to conditions such as endocarditis (71–73), septic arthritis (74, 75), sepsis (76), skin abscesses and ulcers (77, 78), and thrombotic thrombocytopenic purpura-like syndrome (71). However, zoonotic transmission of *T. pyogenes* from animals to humans has not been conclusively demonstrated (79).

Although *T. pyogenes* has been known for decades, its reservoirs and transmission routes remain inadequately studied (79). Infected animals can shed the bacteria through feces, which may contaminate soil or food sources (20). Other animals may be infected through ingesting contaminated soil or feed, or by coming into contact with contaminated husbandry equipment (79). In addition to foodborne, direct, and indirect contact transmission, *T. pyogenes* may be transmitted by vectors such as ticks (*Dermacentor reticulatus*) (80) and biting flies (*Hydrotaea irritans*) (81). A study from Georgia, USA, reported a higher prevalence of *T. pyogenes* on the foreheads of male free-ranging white-tailed deer (WTD; *Odocoileus virginianus*) compared to females, suggesting that behavioral or physiological traits such as antler rubbing and sparring may increase the likelihood of bacterial presence in male deer (48).

*Trueperella pyogenes* exhibits resistance to multiple antimicrobials and antibiotics, including aminoglycosides, β-lactams, macrolides, and tetracyclines. Molecular analyses have identified that *T. pyogenes* carries a variety of antimicrobial resistance (AMR) genes, conferring resistance to antimicrobial drugs. These genes include those resistant to aminoglycosides (*aadA1*, *aadA2*, and *aacC*), β-lactams (*blaP1*), macrolides (*ermB* and *ermX*), sulfonamides (*sul1*), tetracyclines (*tetK, tetL*, *tetM*, *tetN*, *tetW*, *tetZ*, and *tet33*), and trimethoprim (*dfr2a*), highlighting the microbe’s potential for developing antimicrobial resistance (14, 21, 28, 62, 82, 83).

In addition to variability in resistance genes, *T. pyogenes* strains have demonstrated variability in their virulence factors. Virulence is generally defined as the ability of a microorganism to surpass host defenses and cause disease or harm. Virulence factors allow the bacteria to colonize the host at a cellular level by facilitating invasion, disease development, and evasion of the host’s immune system (84). Common virulence factors in *T. pyogenes* include collagen-binding protein (*cbpA*), fimbria (*fimA*, *fimC*, *fimE*, and *fimG*), neuraminidases (*nanH* and *nanP*), and pyolysin (*plo*) (14, 21, 25, 26, 85–87), and the *plo* gene has been consistently detected in all *T. pyogenes* isolates. A study conducted on free-ranging WTD in Georgia, USA, found that virulence genes related to epithelial attachment in *T. pyogenes* were significantly more frequent at sites where individuals tested positive for cranial abscesses, compared to areas without such abscesses (88). This finding suggests that the pathogenic potential of *T. pyogenes* may contribute to the spatial variability of infections (88).

White-tailed deer are among the most widely distributed wildlife species in North America. According to the United States Department of Agriculture 2022 Census of Agriculture, there are 143 deer farms in Florida, ranking the state fifth in the nation for cervid farming as of 2022 (https://www.nass.usda.gov/AgCensus/, accessed on 20 August 2025). The deer farming industry and wildlife populations are increasingly threatened by infectious agents, including bluetongue virus and epizootic hemorrhagic disease virus, which have the potential to cause significant economic losses and ecological disruption (89). In addition to negatively affecting cervid populations, some of the infectious agents may be zoonotic pathogens (90). To better understand infectious diseases affecting farmed WTD in Florida and to support disease prevention efforts, the Cervidae Health Research Initiative (CHeRI) at the University of Florida (UF) has provided on-site necropsy and diagnostic services to deer farms across Florida since 2016.

Although *T. pyogenes* infections have been identified in both farmed and wild WTD in multiple states in the USA, its epidemiology, antimicrobial resistance, and virulence factors have not been well studied in farmed WTD in Florida. Therefore, the objectives of this study were to investigate the demographic patterns, phenotypic and genotypic AMR profiles, and virulence-associated genomic features of *T. pyogenes* isolated from farmed WTD in Florida. By integrating demographic observations with whole-genome sequencing, we aimed to characterize the occurrence and clinical presentations of *T. pyogenes* infection in this population and to further examine the genetic determinants that may contribute to pathogenicity, antimicrobial resistance, and potential transmission patterns. This study provides baseline data on the local diversity and characteristics of *T. pyogenes*, supporting future disease surveillance, antimicrobial stewardship, and farm management efforts at the wildlife-livestock interface.

## MATERIALS AND METHODS

### Necropsy and specimen collection

**Fig 1:**
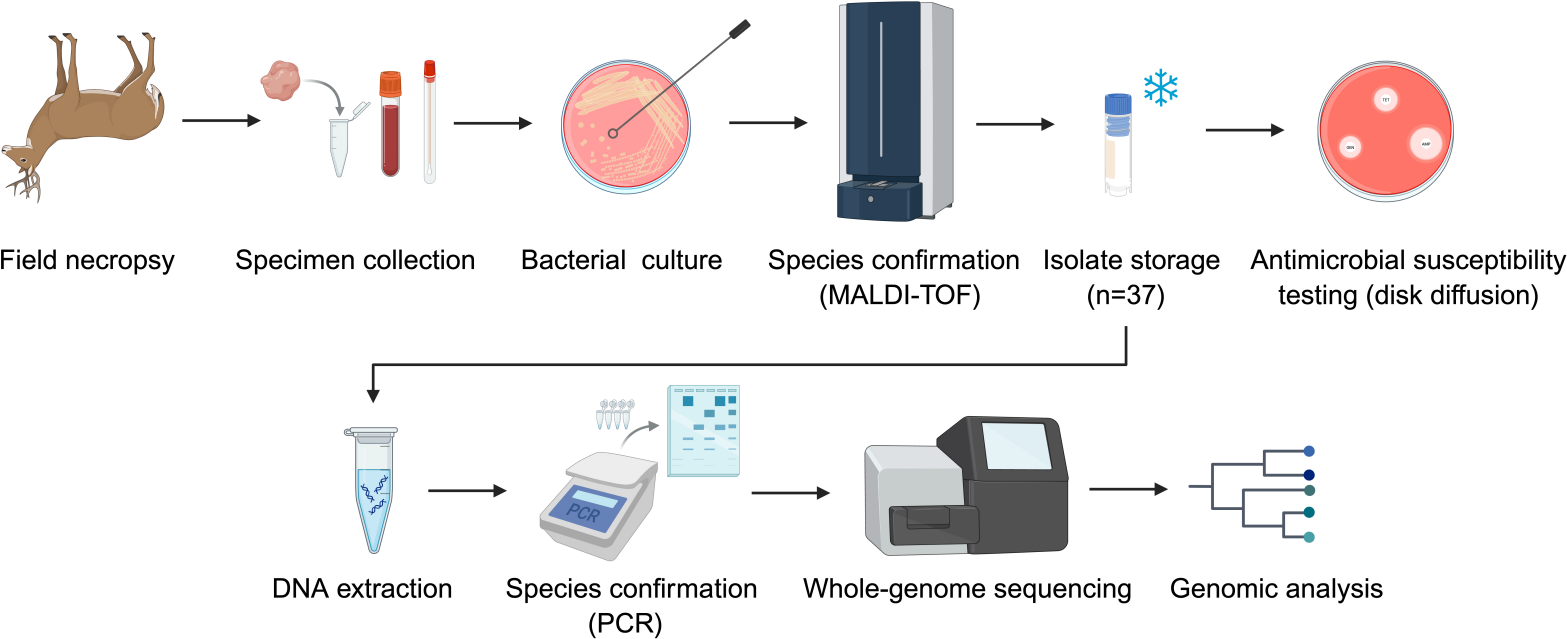
Workflow for isolation and characterization of *Trueperella pyogenes* isolated from deceased farmed white-tailed deer.

The necropsy and specimen collection for this study were carried out by UF CHeRI necropsy team. Necropsies occurred at the behest of deer farmers and were performed on farms. The animal’s age, gross observations, identification, location, and medical history were documented before initiation of a necropsy. Tissue specimens were cut into approximately 1 cm^3^ sizes and placed in 5 mL sterile Eppendorf snap cap tubes (Thermo Fisher Scientific, Waltham, MA, USA). Routinely collected tissue specimens included cardiac tissue (CT), hepatic tissue (HT), kidney tissue (KT), lung tissue (LT), skin tissue (SK), and spleen tissue (ST). Lesion swabs (LS), pus swabs (PS), and scab swabs (SS) were collected using sterile flocked nylon swabs (Copan Diagnostics, Murrieta, CA, USA) for bacteria and viruses collection or CultureSwab Plus Amies Gel (Becton Dickinson, Franklin Lakes, NJ, USA) for bacteria collection. The flocked nylon swabs were placed in empty sterile 15 mL centrifuge tubes (Thermo Fisher Scientific, Waltham, MA, USA), and culture swabs were immersed in the provided gel tubes. During necropsy, photos of the animal and each specimen were captured. All specimens were refrigerated during transportation. The specimens not submitted for bacterial pathogen isolation were stored in a −80°C freezer immediately upon arrival of the necropsy team at the UF College of Veterinary Medicine (CVM). An illustrated workflow of specimen collection, bacterial culture, isolation, and characterization of *T. pyogenes* from a deceased farmed WTD was presented in Fig. 1.

### Descriptive analysis of demographic and case-associated data

Demographic and case-associated data were summarized descriptively to examine the distribution of *T. pyogenes* infection cases across Florida WTD management unit (DMU) areas, seasons, age groups, sex, and clinical presentations (pneumonia and abscesses around the antler pedicle). DMUs are defined by the Florida Fish and Wildlife Conservation Commission (FWC) based on factors such as deer population density, habitat type, hunting regulations, and management objectives. *P* values were calculated using the Chi-square test. The differences were considered statistically significant if the *P* value was <0.05. The DMU map and area range were obtained from FWC (https://geodata.myfwc.com/datasets/myfwc::white-tailed-deer-management-unit-areas-in-florida/about, accessed on 20 August 2025).

### Bacterial culture and isolation

The specimens required for bacterial pathogen isolation were stored under 4°C and then submitted to the UF Microbiology, Parasitology, and Serology Diagnostic Laboratory of the CVM for aerobic or anaerobic bacterial culture, depending on the specimen type and suspicion of bacterial involvement in the lesion. Specimens for bacteriological analysis were cultured onto general blood agar, chocolate agar, Columbia nalidixic acid (CNA) agar, and MacConkey agar plates, which were then incubated under optimal conditions to facilitate the isolation of both aerobic and anaerobic pathogens. Bacterial species identification was performed using a MALDI-TOF (MALDI Biotyper Sirius One System, Bruker, Billerica, MA, USA). The animals were confirmed as positive for *Trueperella pyogenes* infection if the bacterium was isolated from at least one of their specimens.

Thirty-seven *T. pyogenes* isolates cultured from 30 animals between September 2020 and May 2022 were included, representing all isolates recovered during this period without pre-selection (Tables S1 and S2). The *T. pyogenes* colonies were collected from the original CNA agar from the Clinical Microbiology Laboratory and transferred to CryoSavers tubes with Brucella broth and 10% glycerol (Hardy Diagnostics, Santa Maria, CA, USA), using disposable inoculating loops (Thermo Fisher Scientific, Waltham, MA, USA), and were stored in a −80°C freezer for storage. For colony reculture, the colonies were transferred from frozen culture to Columbia agar plates with 5% sheep blood (Thermo Fisher Scientific, Waltham, MA, USA) using sterile toothpicks, and then incubated in an aerobic chamber at 37°C and ambient air for 24 hours. Single colonies were transferred to 5 mL sterile Tryptic Soy Broth + 5% heat-inactivated fetal bovine serum (Catalog number: A3840001, Thermo Fisher Scientific, Waltham, MA, USA) using sterile toothpicks for DNA extraction, and to 5 mL Mueller–Hinton broth (MHB, Thermo Fisher Scientific, Waltham, MA, USA) with 5% heat-inactivated fetal bovine serum (Catalog number: A3840001, Thermo Fisher Scientific, Waltham, MA, USA) for AMR testing as described in the following section.

### Genomic DNA extraction

Extraction of *T. pyogenes* DNA was performed using a Qiagen DNeasy Blood and Tissue kit (Thermo Fisher Scientific, Waltham, MA, USA), following the protocol provided for gram-positive bacteria pretreatment and total DNA purification from animal tissues (spin-column). The concentrations and 260/280 ratios of extracted DNA were measured using a Nanodrop 8000 spectrophotometer (Thermo Fisher Scientific, Waltham, MA, USA).

### Polymerase chain reaction

To confirm the isolated bacterial species, conventional PCR (cPCR) was conducted with 1 μL of DNA input (up to 100 ng per reaction), 12.5 μL of 2× PCR master mix (Promega, Madison, WI, USA), 8.5 μL of water, and 1.5 μL of each primer targeted to the *T. pyogenes*-specific *pyolysin* gene obtained from Eurofins Scientific (Louisville, KY, USA). The forward primer (5′-GGC CCG AAT GTC ACC GC-3′) and the reverse primer (5′-AAC TCC GCC TCT AGC GC-3′) produced an expected amplicon size of 270 bp (91). Thermocycling was performed as follows: 94°C initial denaturation for 2 minutes, 30 cycles of denaturation at 94°C for 1 minute, primer annealing at 55°C for 1 minute, and elongation at 72°C for 1 minute, with a 72°C final elongation for 5 minutes, then 4°C for ∞ (91). PCR amplicons were visualized through gel electrophoresis with 5 μL of the cPCR product mixed with 1 μL of 6× Orange gel dye (Thermo Fisher Scientific, Waltham, MA, USA) and loaded onto a 1% UltraPure agarose gel (Invitrogen, Carlsbad, CA, USA) containing 1× EtBr DNA gel stain (Invitrogen, Carlsbad, CA, USA) and 1× TBE buffer (Thermo Fisher Scientific, Waltham, MA, USA) under 150 V for 30 minutes. A negative PCR control and a positive PCR control were included in each cPCR run.

### Genome sequencing

The bacterial genomic DNA extracted from each sample was pooled together and used to construct a DNA library. This process utilized the Nextera XT DNA Library Preparation Kit (Illumina, San Diego, CA, USA) for the library preparation and the Nextera XT Index Kit (Illumina, San Diego, CA, USA) for sample multiplexing, both of which were carried out according to the manufacturer’s protocol. The DNA library was sequenced using MiSeq v2, 2 × 250 cycle cartridge (Illumina, San Diego, CA, USA) on a MiSeq platform (Illumina, San Diego, CA, USA).

### Sequence analysis

The FASTQ raw sequencing files were uploaded to the Galaxy instance of the University of Florida Research Computing (UFRC) for data processing and analysis (92). The quality and length trimming were conducted using the Galaxy tool Sickle with the quality threshold of 30 and length threshold of 50. The *de novo* genome assembly was performed using the Galaxy tool SPAdes with the K-mers set as 21, 33, 55, 77, 99, 127, and the coverage cutoff set as auto. The core genome alignment and maximum-likelihood phylogenetic tree construction were performed based on core-genome single-nucleotide polymorphisms (SNPs) using Parsnp with the default setting (93). The contigs of 37 isolates were compared with 16 *T. pyogenes* complete genome sequences and whole-genome shotgun sequences from the NCBI GeneBank database. The phylogenetic tree was visually explored using Gingr (93). The reference strains were listed in Table S3. To annotate virulence factors, the assembled genome files were uploaded to CLC Genomics Workbench v2.0 (Qiagen, Germantown, MD, USA) and analyzed using the CLC Microbial Genomics Module. The nucleotide sequences were annotated against the Virulence Factor Database (VFDB) (94) utilizing default parameters. The AMR genes were annotated with the MEGARes database using AMR++ v3.0 (95), and SNP mutants were excluded.

### Antimicrobial susceptibility testing

Since the Clinical and Laboratory Standards Institute (CLSI) guideline does not provide an antimicrobial susceptibility disk diffusion testing (Kirby-Bauer test) protocol for *T. pyogenes*, the protocol was modified from the one for *Streptococcus pneumoniae*, which is also a facultatively anaerobic and fastidious bacterium according to the CLSI M100 guideline (96), as suggested in previous studies (15, 21). Single colonies were inoculated into 5 mL MHB with 5% bovine serum and kept in a rotating shaker in an aerobic chamber at 37°C and ambient air for 24 hours as a seed culture. Five microliters of seed cultures were transferred to 5 mL MHB with 5% bovine serum (Catalog number: A3840001, Thermo Fisher Scientific, Waltham, MA, USA) and kept in a rotating shaker in an aerobic chamber at 37°C for 24 hours as the main culture. After 24 hours of incubation, when the main culture reached the log growth phase as determined by the OD value, 100 µL of the culture was transferred onto Mueller–Hinton agar supplemented with sheep blood (Thermo Fisher Scientific, Waltham, MA, USA) and evenly spread using sterile glass beads. The antimicrobial disks contained ampicillin (10 ug/disk), ceftiofur (30 ug/disk), enrofloxacin (5 ug/disk), gentamicin (10 ug/disk), neomycin (30 ug/disk), oxytetracycline (30 ug/disk), penicillin (10 ug/disk), tetracycline (30 ug/disk), and trimethoprim–sulfamethoxazole (25 ug/disk) (Thermo Fisher Scientific, Waltham, MA, USA) were tamped onto the plates and the plates incubated in an aerobic chamber under 37°C and ambient air for 48 hours. The antibiotics were selected due to their common usage in deer farming and the documented resistance observed in certain *T. pyogenes* strains in previous studies. The incubation period was extended to 48 hours to accommodate the fastidious growth characteristics of *T. pyogenes* in accordance with previously established recommendations (97). The diameters of the zone of inhibition (ZOI) were measured and noted. The ZOI diameter interpretive criteria for *S. pneumoniae* in the CLSI M100 guideline was used to define the ZOI, as suggested in previous studies (15, 21). To evaluate the correlation between the phenotypic and genotypic AMR profiles of *T. pyogenes* strains against the antibiotics (gentamicin, neomycin, oxytetracycline, and tetracycline), inhibition zone measurements and the presence of associated resistance genes (aminoglycosides: *ant3-dprime*, *ant6*, *a16s*, and *rrs*; tetracyclines: *tetR*, *tetW*, *tetZ*, *tet16S*, and *tet33*) were analyzed. Statistical significance was determined using an unpaired *t*-test, with differences considered significant at a *P* value < 0.05.

## RESULTS

### Clinical characteristics and distribution of *Trueperella pyogenes* infections

**Fig 2:**
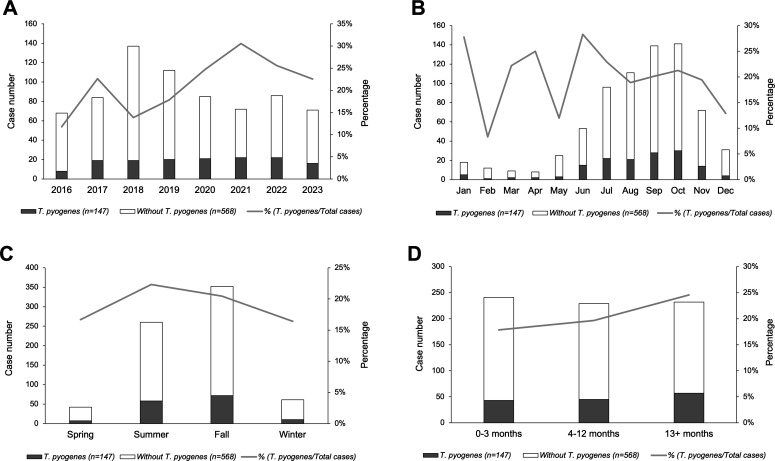
*Trueperella pyogenes* infection cases in farmed white-tailed deer in Florida from 2016 to 2023 (**A**) by sampling year, (**B**) by sampling month, (**C**) by sampling season, and (**D**) by host animal age (*n* = 147). Bar plots represent the case numbers with or without *T. pyogenes* and line plots represent the *T. pyogenes* case percentages out of total necropsy/sampling cases with bacterial culture results (*n* = 715). Seasons were defined as spring (1 March–31 May), summer (1 June–31 August), fall (1 September–30 November), and winter (1 December–28/29 February).

**Fig 3:**
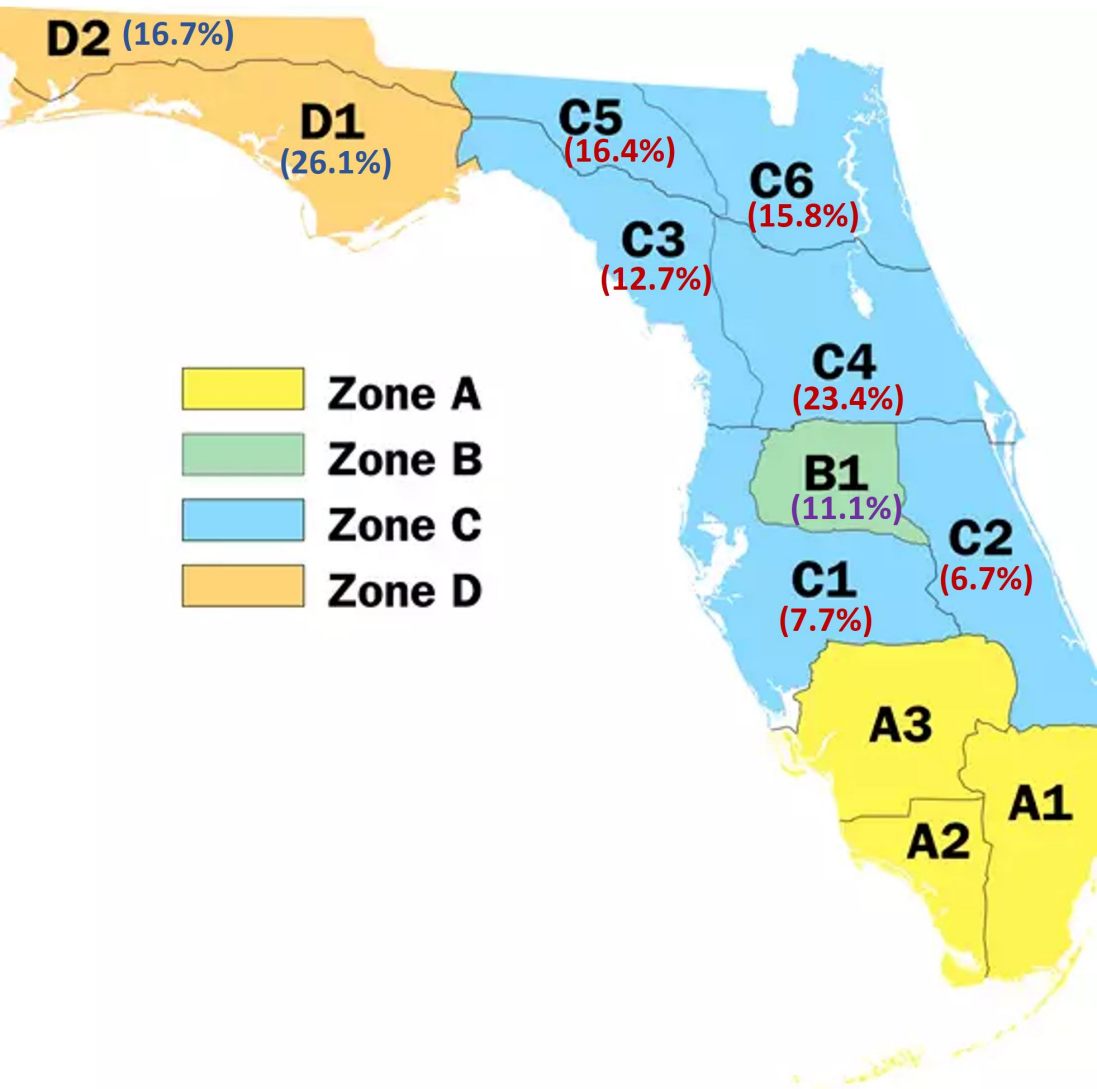
Percentage of *Trueperella pyogenes* infection in farmed white-tailed deer by Florida white-tailed DMU from 2016 to 2023. The numbers represent the percentages of *T. pyogenes* infection cases (*n* = 147) out of total necropsy cases (*n* = 715) in each DMU. Deer management unit map modified from the Florida Fish and Wildlife Conservation Commission (https://geodata.myfwc.com/datasets/myfwc::white-tailed-deer-management-unit-areas-in-florida/about, accessed on 20 August 2025).

Between 2016 and 2023, CHeRI managed 831 cases involving deceased farmed WTD to determine causes of mortality. These cases included both on-site necropsies and analyses of shipped specimens from the owners. Specimens from 715 animals were submitted to the UF Microbiology, Parasitology, and Serology Diagnostic Laboratory of the CVM for aerobic or anaerobic bacterial culture, depending on specimen type and suspicion of bacterial involvement in lesions. The most frequently isolated bacterial pathogens were *Escherichia coli* (*n* = 253, 35.4%), followed by *T. pyogenes* (*n* = 147, 20.6%), *Streptococcus* spp. (*n* = 84, 11.7%), and *Pseudomonas* spp. (*n* = 70, 9.8%) (Fig. S1). Their presence at the time of mortality suggested a potential role in contributing to the observed clinical signs. From 2016 to 2021, both the number and proportion of cases involving *T. pyogenes* infections showed an overall increasing trend (Fig. 2A). Among the 147 cases of *T. pyogenes* infections, many occurred during the summer and fall seasons (Fig. 2B and C). Statistical analyses revealed that the distribution of *T. pyogenes* cases across the different seasons was significantly different (*P* value < 0.0001) (Table 3). However, it should be noted that necropsy cases were also higher during these seasons, leading to a fluctuation in the monthly percentages of *T. pyogenes* cases (Fig. 2B). We did not find a difference in *T. pyogenes* cases among animals for different ages (*P* value = 0.3054) (Fig. 2D; Table 3). The 147 *T. pyogenes* cases were located in 9 DMUs (Fig. 3; Table S4), with the highest case numbers in DMU D1 and C4. The total *T. pyogenes* case numbers among 9 DMUs were significantly different (*P* value < 0.0001) (Table 3). The number of cases involving male deer with *T. pyogenes* was significantly higher than the number of cases involving female deer (*P* value = 0.01) (Table 3). Of the 715 necropsy cases from 2016 to 2023, *T. pyogenes* was isolated from 25.9% (88/340) of males and 15.9% (57/359) of females.

**TABLE 3:** Association of *Trueperella pyogenes* infection cases with season, age, deer management unit, sex, and clinical signs^*a*^

	*T. pyogenes* infection cases, whose isolates were analyzed in this study (*n* = 30)		Total *T. pyogenes* infection cases (*n* = 147)	
Factors	Case number	*P* value	Case number	*P* value
Season		<0.0001***		<0.0001***
Spring	4		7	
Summer	1		58	
Fall	22		72	
Winter	3		10	
Age		0.0074**		0.3054
1–3 months	5		43	
4–12 months	18		45	
13+ months	7		57	
DMU area		<0.0001***		<0.0001***
B1	0		1	
C1	0		2	
C2	1		3	
C3	0		8	
C4	2		29	
C5	5		10	
C6	2		9	
D1	19		84	
D2	1		1	
Sex		0.0679		0.01*
M	20		88	
F	10		57	
Pneumonia		<0.0001***		
Yes	30		–^*b*^	
No	0		–	
Antler infection		0.0029**		
Yes	3		–	
No	16		–	
N/A	11		–	

^*a*^ Season definitions: spring: 1 March–1 June; summer: 1 June–1 September; fall: 1 September–1 December; winter: 1 December–1 March. DMU, deer management unit. *P* value significance: *P* values are indicated with stars as follows: *P* value < 0.05 (*), *P* value < 0.01 (**), *P* value < 0.001 (***).

^*b*^ – Not available.

**Fig 4:**
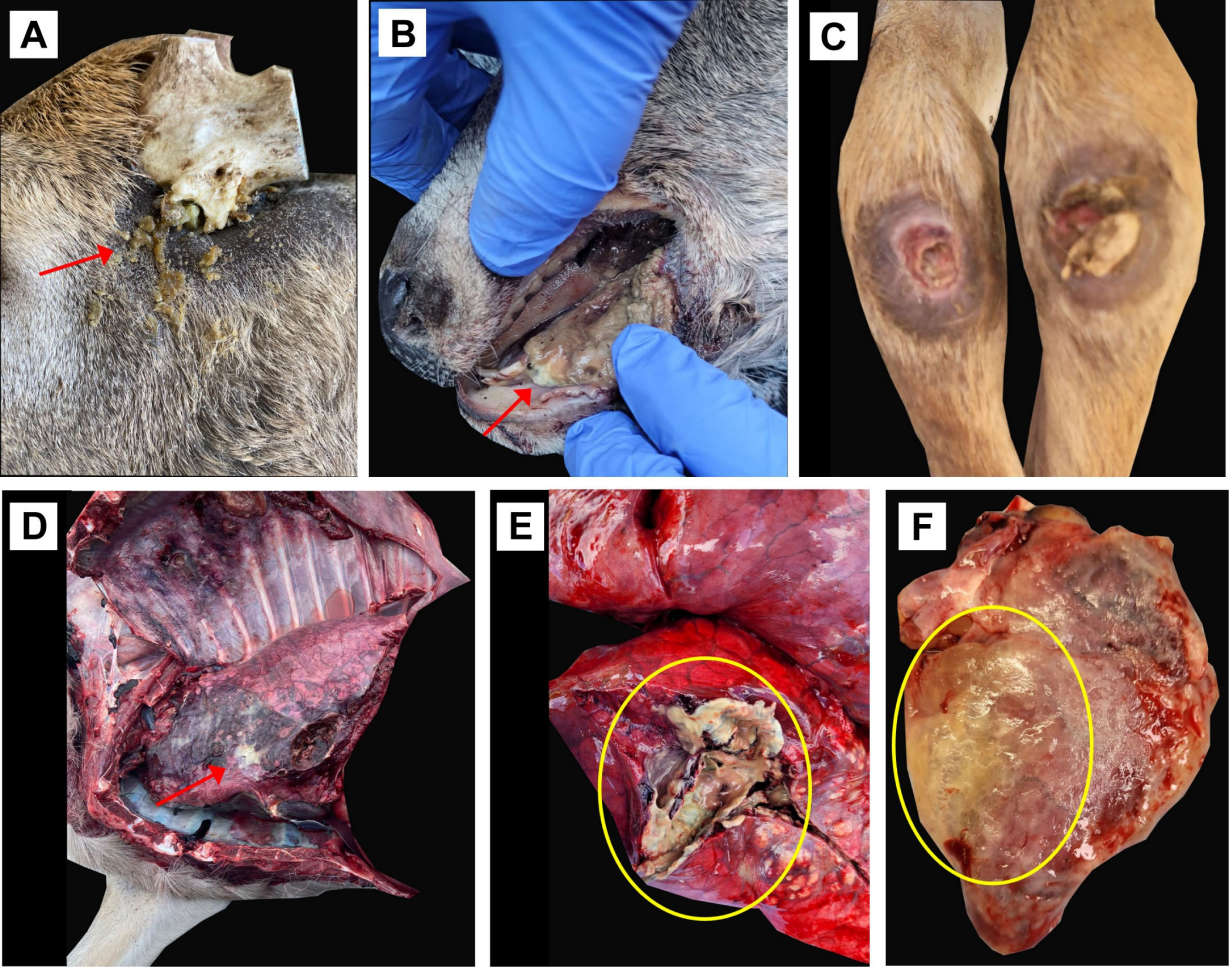
Gross observation of the *Trueperella pyogenes* lesions in farmed white-tailed deer in Florida. (**A**) Lesions and pus at the base of the antler pedicle of OV1430. (**B**) Oral cavity with lesions and purulent discharge in the gums and tongue of OV1615. (**C**) Open wound with associated purulent discharge of OV1601. (**D**) Discolored and hemorrhaging lungs with pus of OV1625. (**E**) Pus within pulmonary tissue from OV1492. (**F**) Heart with pericardium exhibiting a layer of white and yellow purulent material of OV1504.

In this study, 37 *T. pyogenes* isolates (Table S2) were collected from various specimen types across 30 animals (Table S1) between September 2020 and May 2022. Among the 30 animals from which *T. pyogenes* was isolated, all presented signs of pneumonia. The majority of the animals exhibited gray and pale discoloration in the lungs, with some also showing abscess formation and pleural effusion within the chest cavity (Fig. 4). Three of the animals (deer OV1430, OV1565, and OV1570) had lesions with pus at the base of the antler pedicle and *T. pyogenes* was isolated from the antler lesion swab of OV1430. Three animals presented jaw and oral cavity lesions. Specifically, deer OV1458 exhibited a lumpy jaw with food impaction, while OV1459 had a partially missing lower jaw, and OV1615 presented with lesions and purulent discharge affecting the gums and tongue. Bacterial species other than *T. pyogenes* were cultured from 25 specimens obtained from 21 of the 30 animals, including *Escherichia coli*, *Streptococcus uberis*, and *Pseudomonas aeruginosa* (Table S2).

### Genome assembly and phylogenetic analysis

All genome assemblies have been deposited in NCBI GenBank under the BioProject PRJNA1338263. Genome assembly statistics for the 37 *T. pyogenes* isolates are shown in Table S5. The average genome size was 2,302,731 ± 10,042 (SE) bp, with a mean G+C content of 59.56% ± 0.02%. On average, the assemblies consisted of 32 ± 2 contigs, with an N50 of 313,157 ± 33,876 bp and a largest contig length of 536,980 ± 41,775 bp.

**Fig 5:**
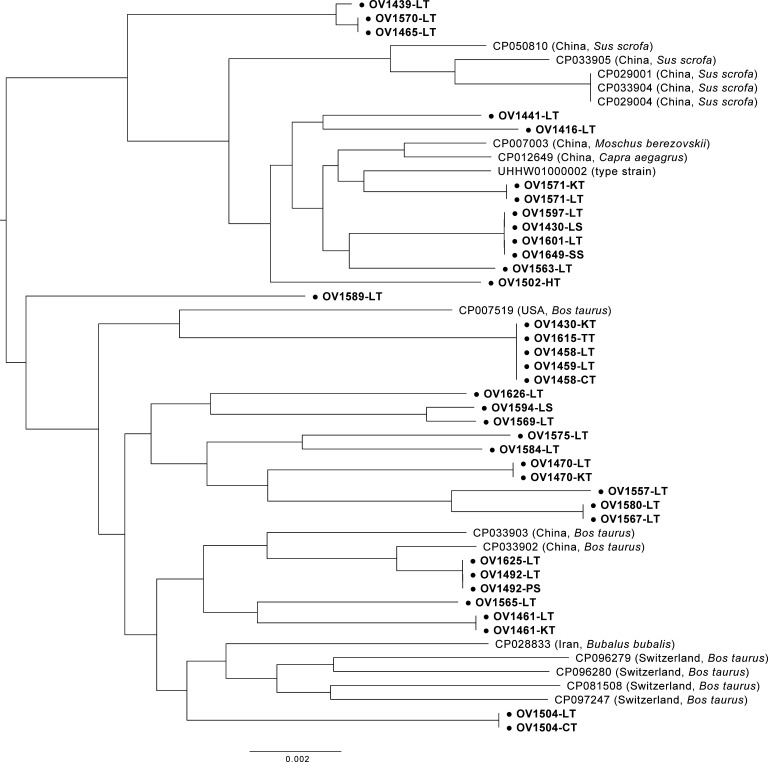
Core-genome SNP phylogeny of the 37 *Trueperella pyogenes* strains from this study and 16 reference strains from NCBI GenBank. The maximum-likelihood phylogenetic tree was constructed based on core-genome SNPs using Parsnp v1.2. Strains from this study are indicated by filled circles (●) and are shown in bold, whereas reference strains obtained from NCBI GenBank are shown without symbols in regular font. Host species and country of origin are indicated in parentheses following each GenBank accession number. Details of the reference strains are provided in Table S3.

The Parsnp core genome alignment used for phylogenetic analysis consisted of 1,807,539 bp. The phylogenetic tree constructed based on core-genome SNPs is illustrated in Fig. 5. This analysis identified two clusters. The first cluster comprised reference sequences from small artiodactyls, including pigs and sheep. Conversely, the second cluster contained reference sequences from large artiodactyls, such as cattle and buffalo. However, the *T. pyogenes* strains isolated from WTD in this study were distributed across both clusters and did not exhibit phylogenetic proximity based on host species, unlike the reference strains. All strains from the same animals cluster together except isolates from OV1430. The WTD strains did not cluster together based on animal location, specimen types, age, sex, or medical history.

### Virulence factor genes

**Fig 6:**
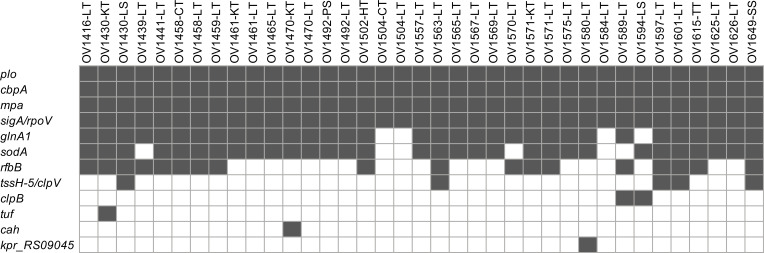
Presence/absence profile of annotated virulence factor genes in the 37 *Trueperella pyogenes* strains. The antimicrobial-resistant genes of 37 *T. pyogenes* strains were identified by comparing their whole-genome sequences against the VFDB. The genomic features for virulence factor genes are represented by their presence (gray square) or absence (white square).

The annotated virulence factors identified in the 37 *T. pyogenes* isolates are shown in Fig. 6, while the class and mechanism of the genes were presented in Table S6. Virulence factor genes encoding for collagen binding protein (*cbpA*), proteasome ATPase (*mpa*), pyolysin (*plo*), and RNA polymerase sigma factor (*sigA*/*rpoV*) were identified in the genomes of all strains. Virulence genes encoding type I glutamate ammonia ligase (*glnA1*) (*n* = 33, 89.2%) and manganese superoxide dismutase (*sodA*) (*n* = 31, 83.8%) were identified in the majority of the genomes from the *T. pyogenes* strains. Other genes encoding dTDP-glucose 4,6-dehydratase (*rfbB*) (*n* = 18, 48.6%), Clp-type ATPase chaperone protein (*tssH-5*/*clpV*) (*n* = 5, 13.5%), type VI secretion system ATPase ClpV1 (*clpB*) (*n* = 2, 5.4%), elongation factor Tu (*tuf*) (*n* = 1, 2.7%), Ag43/Cah family autotransporter adhesin (*cah*) (*n* = 1, 2.7%), and NAD-dependent epimerase (*KPR_RS09045*) (*n* = 1, 2.7%) were sporadically presented in the genome of a few strains. The presence of bacterial virulence factors was not correlated with animal location, specimen types, age, sex, or medical history.

### Antimicrobial resistance genes

**Fig 7:**
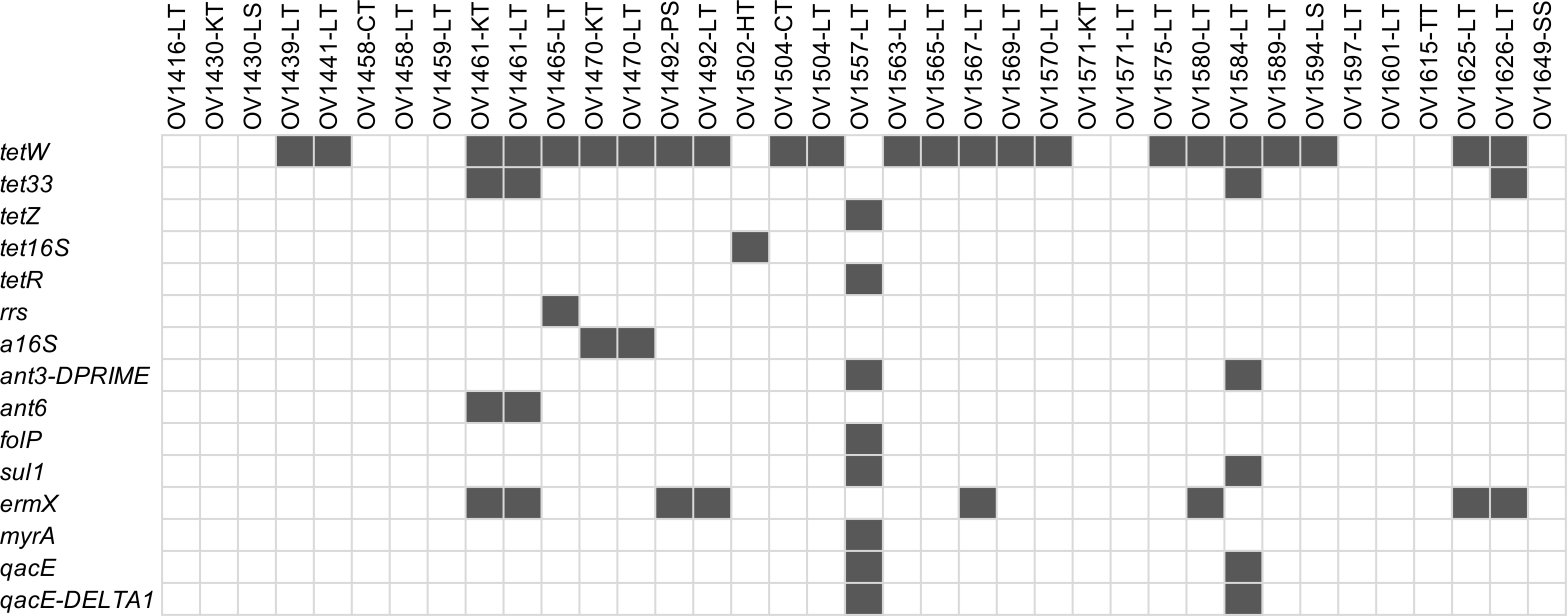
Presence/absence profile of annotated antimicrobial resistance genes in the 37 *Trueperella pyogenes* strains. The antimicrobial-resistant genes of 37 *T. pyogenes* strains were identified by comparing their whole-genome sequences against the MEGARes database v3.0 using the AMR++ pipeline and SNP mutants were excluded. The genomic features for antimicrobial resistance are represented by their presence (gray square) or absence (white square).

The annotated AMR genes among the 37 *T. pyogenes* isolates are shown in Fig. 7, with detailed classifications and mechanisms of these genes provided in Table S7. An AMR gene for tetracycline resistance (*tetW*) was the most prevalent, identified in 23 (62.2%) strains. Other AMR genes were detected sporadically, including those conferring resistance to aminoglycosides (*ant3-dprime*, *ant6I*, *a16S*, and *rrs*), sulfonamides (*folP* and *sul1*), and tetracyclines (*tetR*, *tetZ*, *tet16S*, and *tet33*). Notably, 12 (32.4%) strains lacked any detectable AMR genes. Twenty-five (67.6%) strains carry at least one of the tetracycline resistance genes (*tetR*, *tetW*, *tetZ*, *tet16S*, or *tet33*). No correlation was observed between the presence of AMR genes and factors such as animal location, specimen type, age, sex, or medical history.

### Antimicrobial-resistant disk diffusion testing

The disk diffusion test results are presented in Table S8. The ZOI diameters for tetracycline and oxytetracycline exhibited the greatest variability among the 37 strains compared to other antimicrobials. Based on the ZOI diameter interpretive standard for *Streptococcus pneumoniae* in the CLSI M100, none of the *T. pyogenes* strains were resistant to penicillin (*n* = 0, 0%), and most of the strains were resistant to trimethoprim-sulfamethoxazole (*n* = 36, 97.3%) and tetracycline (*n* = 34, 91.9%). All tetracycline-sensitive and intermediate strains lacked detectable tetracycline resistance genes, and nine tetracycline-resistant strains were also found to lack these resistance genes.

**Fig 8:**
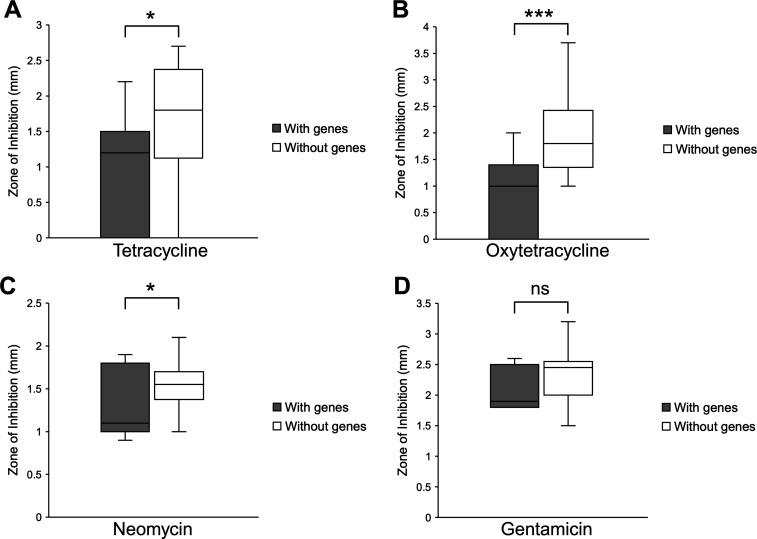
Association between the zone of inhibition and the presence of antimicrobial resistance genes for (**A**) tetracycline (*tetW*, *tet33*, *tetZ*, *tet16S*, and *tetR*), (**B**) oxytetracycline (*tetW*, *tet33*, *tetZ*, *tet16S*, and *tetR*), (**C**) neomycin (*rrs*, *a16s*, *ant3-dprime*, and *ant6*), and (**D**) gentamicin (*rrs*, *a16s*, *ant3-dprime*, and *ant6*). Strains with at least one antimicrobial resistance gene corresponding to each antibiotic are categorized under the “With genes” group. Statistical significance is denoted as follows: *P* value < 0.05 (*), *P* value < 0.01 (**), *P* value < 0.001 (***), and *P* value > 0.05 (ns).

Based on the AMR disk diffusion testing and gene annotation results, the zones of inhibition for tetracycline were significantly smaller (*P* value = 0.0144) in strains harboring tetracycline resistance genes (*tetR*, *tetW*, *tetZ*, *tet16S*, or *tet33*) compared to those without these genes. Similarly, the ZOI for oxytetracycline was significantly smaller (*P* value < 0.0001) in strains carrying tetracycline resistance genes. Strains carrying aminoglycoside resistance genes (*ant3-dprime*, *ant6I*, *a16S*, or *rrs*) exhibited significantly smaller ZOI around disks containing neomycin (*P* value = 0.04) compared to those lacking these genes. However, the presence of aminoglycoside resistance genes did not result in a significant difference in the ZOI diameter around the gentamicin-containing disks when compared to strains lacking these resistance genes (*P* value = 0.0783) (Fig. 8).

## DISCUSSION

### Distribution of *Treuperella pyogenes* infections

*Treuperella pyogenes* has been identified as an opportunistic pathogen and zoonotic agent for decades. *T. pyogenes* has been extensively studied in cattle and swine (Tables 1 and 2), but research on its occurrence in other artiodactyls, such as deer, was markedly limited. This study is the first report of *T. pyogenes* in Florida farmed WTD. These genome-based characteristics of *T. pyogenes* provide a baseline for monitoring and comparing the distribution of this pathogen over time in populations of farmed deer in Florida. The approximate time period wherein *T. pyogenes* became established as a pathogen for Florida’s WTD is uncertain due to the lack of previous research. However, our data indicate a rise in case numbers of *T. pyogenes* infection since 2016. Given its broad host range and the increasing proportion of cases observed in farmed WTD, *T. pyogenes* should be considered a potential emerging pathogen, warranting further research and targeted preventive measures.

In contrast to seasonal hemorrhagic viral disease outbreaks in Florida, which typically occur from August to October (89), *T. pyogenes* infections tend to rise in June but continue to occur sporadically throughout the winter and spring. The seasonal pattern of *T. pyogenes* infections in Florida farmed WTD can be attributed to several factors. Temperature itself can affect the growth of *T. pyogenes*. The optimum growth temperature for *T. pyogenes* is 37°C (98), which aligns with typical summer conditions in Florida, beginning in June. Warmer temperatures enable *T. pyogenes* to persist and proliferate in the environment following shedding, increasing the likelihood of environmental transmission. *T. pyogenes* primarily spreads through direct contact or indirectly via contaminated soil or water, rather than through vector-borne transmission (20, 79–81). The results supported that the transmission of *T. pyogenes* is not primarily reliant on vector insects, which are typically active during warmer months and less common in colder seasons. As a result, *T. pyogenes* transmission can sporadically continue even in cooler seasons.

Although the difference in case numbers between age groups was not statistically significant, yearling animals (over 13 months old) showed a slight tendency toward more *T. pyogenes* infections compared to younger animals. This trend may be attributed to the chronic nature of *T. pyogenes*-related infections (18, 56, 99–101). Unlike other common clinical signs in WTD, such as organ hemorrhage, this condition tends to develop gradually, potentially leading to delayed mortality. Northern Florida had significantly more cases involving infection of WTD with *T. pyogenes* than what occurs in Southern Florida. This discrepancy may be due to a higher number of deer farms in Northern Florida collaborating with CHeRI, which could introduce sampling bias.

The incidence of *T. pyogenes* infections among necropsy cases was higher in male deer compared to females, possibly due to antler infections and trauma during the rut season. A previous study on free-ranging WTD in Georgia, USA, found a higher prevalence of *T. pyogenes* along the foreheads of males, especially in areas with a high occurrence of cranial or intracranial abscess disease (48, 88). However, on deer farms with good management practices, antlers are often removed from male deer, and antler infections are less common. It is noteworthy that all 37 animals in this study exhibited clinical signs of pneumonia, and only three animals presented antler lesions. This result is consistent with the fact that the *T. pyogenes* strains in this study encode different patterns of virulence factors than previous studies, which will be discussed in subsequent sections. Moreover, pneumonia is more challenging to detect through gross observation compared to antler infections, because deer may not show obvious clinical signs until the disease has progressed to an advanced stage. This delay in recognition complicates treatment and management efforts. Additionally, multiple causes of death can coexist, and an initial infection by one pathogen may compromise the host’s immune system, thereby increasing susceptibility to co-infections. As such, it is crucial to explore the relationships among various pathogens to better understand their impacts on health and disease progression in farmed WTD.

### Phylogenetic analysis

For the reference strains from artiodactyl species other than WTD, the core-genome-based SNP phylogenetic tree was divided into two clades based on the host species, suggesting a potential for host-specific adaptation. Previous studies presented similar findings, where *T. pyogenes* strains tended to cluster according to their host species (83, 102). However, the strains isolated from the farmed WTD in this study were polyphyletic among the whole phylogenetic tree, and didn’t cluster together based on the animal location, specimen types, age, sex, or medical history. The greater genetic heterogeneity observed among the *T. pyogenes* strains in this study suggests that multiple genetically distinct lineages may be circulating in farmed WTD in Florida, which may reflect diverse evolutionary origins or introductions, although additional sampling across host species would be required to assess host range or cross-species transmission. Notably, strains OV1430-LS, isolated from the antler lesion swab, and OV1430-KT, from kidney tissue, did not cluster together like other strains from the same individual. Animal OV1430 presented clinical signs, including antler lesions, pneumonia, and friable kidneys, without consistency. These findings suggest that the deer population can harbor multiple strains of *T. pyogenes* from different organ systems, where they may contribute to the complexity of clinical presentations.

### Virulence factors

The whole-genome sequencing approach enabled us to examine all virulence factors in the *T. pyogenes* strains and minimized the limitation of PCR approaches, which can only focus on targeted genes. The *plo* gene, which encodes pyolysin, was found in all *T. pyogenes* strains in this study. This finding is consistent with previous reports, where the *plo* gene was present in all strains and has been used as a marker gene for *T. pyogenes* identification. It is worth noting that the other most prevalent virulence factors identified in the *T. pyogenes* strains in this study differ from those in previous studies. Primarily, the virulence factor *cbpA* was detected in all strains in this study. The gene *cbpA* encodes collagen-binding protein A, which is capable of binding type I collagen (103) and attaching to collagen-rich tissue, such as lung tissue (79, 104). However, *cbpA* is relatively rare in bovine-origin *T. pyogenes* strains, which is mainly related to mastitis and metritis in cattle, instead of pneumonia (8, 14, 20, 105, 106). In contrast, another study found that all of the *T. pyogenes* strains isolated from abscesses in various organs of the forest musk deer carried the *cbpA* gene (86). In a swine pneumonia study, 66.7% of the *T. pyogenes* strains isolated from lung tissues carry the *cbpA* gene (63). A study on *T. pyogenes* in free-ranging WTD with cranial abscess disease revealed that more *T. pyogenes* strains isolated from affected sites carried virulence factor genes, such as *nanH*, *nanP*, *fimA*, *fimE*, and *fimG,* compared to strains from unaffected sites. However, the prevalence of the *cbpA* gene did not differ between the affected and unaffected sites (88). On the other hand, no neuraminidase (*nanP*, *nanH*) or fimbria genes (*fimA*, *fimC*, *fimE*, *fimG*), which are common in bovine-origin *T. pyogenes* strains, were detected in the *T. pyogenes* in this study. These results suggest that *T. pyogenes* isolates involved in different clinical presentations and host species may be differentially equipped with virulence factors that lead to different pathogenicity.

From the perspective of evolutionary biology and virulence trade-off theory, pathogens tend to evolve to optimize their fitness, which corresponds to an optimal level of virulence (107, 108). Increased virulence does not always enhance pathogen fitness. Additionally, farm management practices can influence host condition and exert selection pressure on strains and virulence factors leading to more apparent clinical signs, such as antler infections or mastitis, while maintaining those associated with pneumonia (48, 88). From the viewpoint of microbial ecology, particularly for opportunistic pathogens, host intrinsic factors, the synergism between *T. pyogenes* and other bacteria, and the differential gene expression of virulence factor genes altogether may play a more relevant role in the establishment of infections (109). However, all specimens in this study were collected from deceased animals, either animals with severe clinical signs that died naturally or with more moderate clinical signs and then euthanized by the owners. The *T. pyogenes* strains with lower pathogenicity might not be included in this study. Expanding specimen sources to include animals harvested by hunters, road-killed animals, and farmed deer displaying mild clinical signs should be considered in future research. This wider range of specimen sources could provide a more comprehensive understanding of the prevalence and diversity of *T. pyogenes* infections across different populations and environments.

### Antimicrobial resistance

Like virulence factors, the whole-genome sequencing approach enabled us to examine all AMR genes in the *T. pyogenes* strains and minimize the limitation of PCR approaches, which can only focus on targeted genes. The genome-based AMR profile observed in this study was broadly consistent with previous reports (14, 83, 102, 110), with tetracycline resistance genes being the most common, other AMR genes occurring sporadically, and some strains lacking detectable AMR genes. Several factors may explain this finding. First, tetracycline resistance has been frequently reported in *T. pyogenes* in previous studies (14, 111, 112), indicating that tetracycline resistance genes may already be widespread in circulating strains. Second, the resistance observed in the present study may reflect historical or indirect antimicrobial selection pressures rather than treatment specifically associated with pneumonia. In addition, tetracycline resistance genes are often carried on mobile genetic elements and may persist through horizontal gene transfer or co-selection with other antimicrobial resistance genes (113–116). Therefore, the high frequency of tetracycline resistance observed here may represent broader population- or environment-level selection rather than direct antimicrobial exposure in the individual pneumonia cases examined.

Due to the absence of specific CLSI guidelines for ZOI diameter interpretive criteria for *T. pyogenes*, the criteria established for *Streptococcus pneumoniae*, which is also facultatively anaerobic and fastidious, were utilized in this study. However, this standard may not fully align with the actual resistance profile of *T. pyogenes*, potentially contributing to discrepancies between the observed AMR genotypes and phenotypes, particularly for strains that exhibited intermediate resistance. However, the overall trends in genotypic and phenotypic resistance to tetracycline, oxytetracycline, and neomycin are consistent, indicating the potential effectiveness of molecular approaches for predicting antimicrobial resistance.

### Prevention of *Trueperella pyogenes* infection and deer farm management

Based on the phylogenomic data, *T. pyogenes* in farmed WTD in Florida may have multiple origins. This underscores the critical importance of comprehensive farm management to mitigate disease transmission. Currently, no commercially available vaccines or phage therapies have proven to be effective against *T. pyogenes* (117, 118). Implementation of appropriate management strategies, including maintaining good hygiene, implementing stringent biosecurity measures, preventing interactions between livestock and wildlife, ensuring feed and water sources are uncontaminated, controlling pests, isolating infected animals, and conducting regular disease monitoring, is essential for preventing *T. pyogenes* infections in both captive animals and nearby wildlife. Additionally, considering that *T. pyogenes* is not the only common pathogen associated with pneumonia in WTD, the selection of antimicrobials should account for potential co-infections.

There are some limitations in this study. First, clinical specimens evaluated in this study for *T. pyogenes* positivity were submitted from deceased WTD; it is likely to overestimate the overall prevalence of the post-mortem clinical specimens, and could not perform longitudinal sampling of individual animals over time. Secondly, there is a lack of CLSI disk diffusion standards for *T. pyogenes*, making it difficult to monitor the pathogen in some clinical laboratories (119). Finally, the isolates in this study were obtained from culture-based diagnosis, making it prone to selection bias and lacking the metagenomics data for polymicrobial infections (109).

### Conclusion

In summary, we provided comprehensive insights into the post-mortem prevalence, antimicrobial resistance, and virulence factors of *T. pyogenes* in farmed WTD in Florida. Genomic analyses revealed considerable diversity among the *T. pyogenes* strains, with significant implications for disease transmission and control. The presence of multiple AMR genes underscores the need for cautious antimicrobial use and the development of targeted management strategies to mitigate the risk of spreading resistant strains. Moreover, the identification of key virulence factors highlights the adaptive mechanisms of this pathogen, which may facilitate its persistence and pathogenicity in deer populations. Effective monitoring and preventive measures are crucial in managing *T. pyogenes* infections at the wildlife-livestock interface, thereby protecting both farmed and wild deer populations from potential outbreaks. Future research should expand the scope of specimen collection to include diverse sources, such as hunter-harvested and road-killed animals, to enhance our understanding of *T. pyogenes* epidemiology in free-ranging populations and improve disease management practices.

## Data Availability

All genome assemblies have been deposited in NCBI GenBank under the BioProject PRJNA1338263.

## SUPPLEMENTAL MATERIAL

Figure S1 spectrum.00119-26-s0001.pdf Number of cases of the most frequently isolated bacterial pathogens from farmed white-tailed deer in Florida from 2016 to 2023 (*n* = 715).

Supplemental tables spectrum.00119-26-s0002.xlsx Tables S1 to S8.

ASM does not own the copyrights to Supplemental Material that may be linked to, or accessed through, an article. The authors have granted ASM a non-exclusive, world-wide license to publish the Supplemental Material files. Please contact the corresponding author directly for reuse.
