# Supplementary material for: Genome features and antimicrobial resistance of Trueperella pyogenes isolated from deceased farmed white-tailed deer (Odocoileus virginianus) in Florida
Source: Microbiol Spectr. 2026 Jun 30;14(8):e00119-26. doi: 10.1128/spectrum.00119-26 (PMC13436335; doi:10.1128/spectrum.00119-26)
Supplement: Supplementary file 1 [file spectrum.00119-26-s0001.pdf]

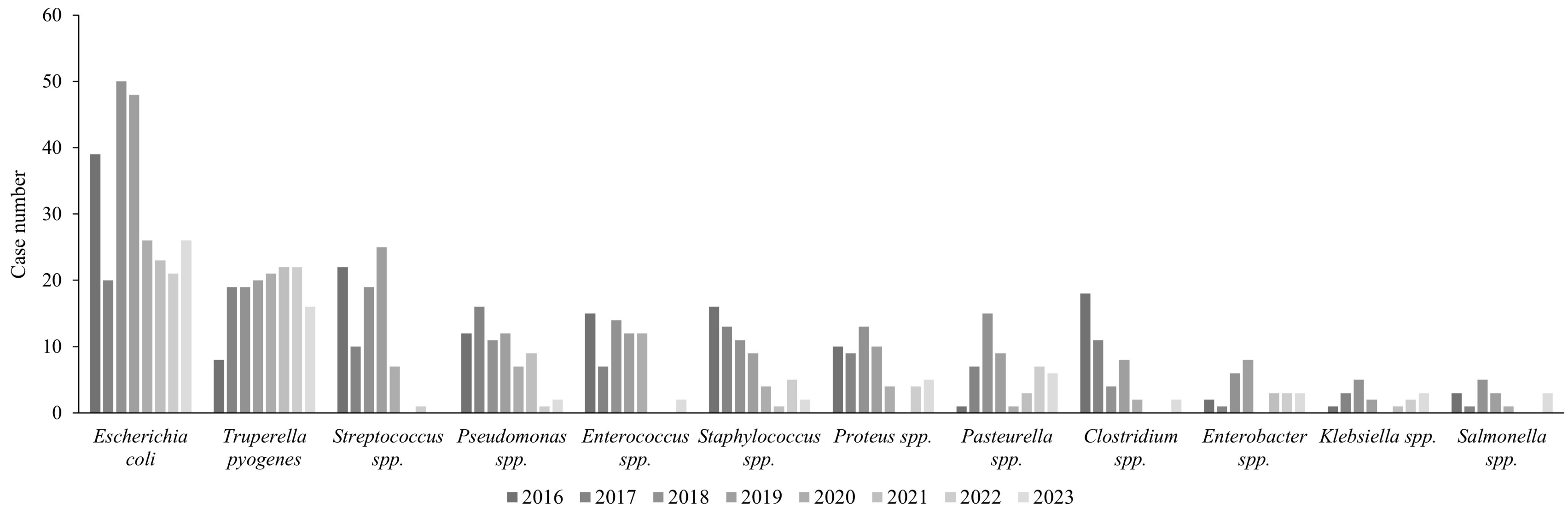

Figure S1. Case number of the most frequently isolated bacterial pathogens from farmed white-tailed deer in Florida from 2016 to 2023 (n = 715).
